# Processing the Loblolly Pine PtGen2 cDNA Microarray 

**DOI:** 10.3791/1182

**Published:** 2009-03-20

**Authors:** W. Walter Lorenz, Yuan-Sheng Yu, Marta Simões, Jeffrey F. D. Dean

**Affiliations:** Warnell School of Forestry and Natural Resources, University of Georgia; Instituto de Biologia Experimental e Tecnológica, Instituto Tecnologia Química e Biológica UNL, Av. da República

## Abstract

PtGen2 is a 26,496 feature cDNA microarray containing amplified loblolly pine ESTs.  The array is produced in our laboratory for use by researchers studying gene expression in pine and other conifer species.  PtGen2 was developed as a result of our gene discovery efforts in loblolly pine, and is comprised of sequences identified primarily from root tissues, but also from needle and stem.^1,2^  PtGen2 has been tested by hybridizing different Cy-dye labeled conifer target cDNAs, using both amplified and non-amplified indirect labeling methods, and also tested with a number of hybridization and washing conditions.  This video focuses on the handling and processing of slides before and after pre-hybridization, as well as after hybridization, using some modifications to procedures developed previously.^3,4^  Also included, in text form only, are the protocols used for the generation, labeling and clean up of target cDNA s, as well as information on software used for downstream data processing.

PtGen2 is printed with a proprietary print buffer that contains high concentrations of salt that can be difficult to remove completely.  The slides are washed first in a warm SDS solution prior to pre-hybridization.  After pre-hybridization, the slides are washed vigorously in several changes of water to complete removal of remaining salts.  LifterSlips™ are then cleaned and positioned on the slides and labeled cDNA is carefully loaded onto the microarray by way of capillary action which provides for even distribution of the sample across the slide, and reduces the chance of bubble incorporation.  Hybridization of targets to the array is done at 48°C in high humidity conditions. After hybridization, a series of standard washes are done at 53°C and room temperature for extended times.  Processing PtGen2 slides using this technique reduces salt and SDS-derived artifacts often seen when the array is processed less rigorously.  Hybridizing targets derived from several different conifer RNA sources, this processing protocol yielded fewer artifacts, reduced background, and provided better consistency among different experimental groups of arrays.

**Figure:**
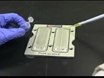


## Protocol

(Note  Sections 1 - 4 Not Demonstrated in Video)

### Preparing Cy-Labeled Target cDNA

What follows is the protocol we use for cDNA synthesis from loblolly pine total RNA and indirect cDNA labeling prior to microarray hybridizations.  Although a few non-trivial modifications have been made, the protocol below is similar to that in the instruction manual provided with the Invitrogen’s SuperScript Indirect cDNA Labeling Kit.

### Part 1: First-Strand cDNA Synthesis Reaction

Mix and briefly spin each kit component before use.
Prepare reactions as follows:
  Xµl DEPC-H_2_O
  Xµl total RNA, 20 µg/reaction (pretreated with Ambion’s Turbo DNase)
  2µl Anchored Oligo(dT)_20_ Primer (2.5µg/µl)
  1µl Random Hexamer Primer
  Total Volume = 18µl
Incubate tubes at 70°C for 5 minutes, and then place on ice for 1-2 minutes.
Add the following to each reaction tube on ice:
  6µl 5X First-Strand buffer
  1.5µl 0.1M DTT
  1.5µl 10mM dNTP mix
  1µl RNaseOUT (40U/µl)
  2µl SuperScript III RT (400U/µl)
Mix gently and spin briefly. Incubate tube overnight at 45°C.
Add 15µl of 1M NaOH to each reaction tube and mix thoroughly.
Incubate tube at 70°C for 10 minutes.
Add 15µl of 1M HCl; mix gently.
Add 20µl 3M sodium acetate (pH 5.2); mix gently.

### Part 2: Purifying First-Strand cDNA.

Add 500µl of Loading Buffer to the cDNA (from Step 1.9) and mix well.
Place a SNAP Purification Column (supplied with the Indirect Labeling kit) on a collection tube and load your cDNA on the column.
Spin at 14,000g at room temperature for 1 minute; discard the flow-through.
Place the SNAP Column onto the same collection tube and add 500µl of Wash Buffer.
Spin at 14,000g at room temperature for 1 minute; discard the flow-through.
Repeat Steps 2.4 and 2.5 three times, for a total of three 500µl washes.
Spin one more time at 14,000g at room temperature for 1 minute sec; discard the flow-through.
Place the SNAP Column onto a new 1.5ml tube.
Add 50µl of warm DEPC-water (50°C) to the SNAP Column and incubate column at room temp for 1 minute. Elute the cDNA via a spin at 14,000g at room temp for 1 minute.
Repeat Step 2.9, using 100µl of warm DEPC-water and the same 1.5ml tube for the eluent.
Add 20µl of 3M sodium acetate (pH 5.2) to the eluent from steps 2.9 and 2.10.
Add 3µl of glycogen (20mg/ml) to the tube and mix.
Add 500µl of ice-cold 100% EtOH, mix well, and incubate the tube for 1 hour at –80°C.
Spin the tube at 14,000g at 4°C for 20 minutes.  Carefully remove the supernatant.
Add 500µl of ice-cold 70% EtOH and spin the tube at 14,000g at 4°C for 5 minute.  Carefully remove the supernatant.
Air-dry the sample for 5 minutes; ensure that all EtOH is removed.
Warm the 2X Coupling Buffer at 37°C for 5 minutes and re-suspend the cDNA sample in 5µl of warm 2X Coupling Buffer.
Heat the cDNA/Coupling buffer at 50°C for 10 minutes and vortex well.  Ensure that your cDNA pellet is fully re-suspended in the 2X Coupling Buffer.

### Part 3: Labeling with Fluorescent Dye

*When preparing the reaction, be careful to minimize exposure of the dye solution to light.  Also, DMSO is hygroscopic and will absorb moisture from the air, which will react with the NHS ester of the dye and significantly reduce the coupling reaction efficiency. Keep the DMSO supplied in the kit in an amber screw-capped vial at –20°C, and let the vial warm to room temperature before opening to prevent condensation.  Use only the DMSO provided with this kit.*

Open one packet of Cy-3 or Cy-5 Dye and add 75µl of DMSO directly to the dye vial.
Add 5µl of the DMSO/dye solution to the tube from Step 2.18.
Mix well and incubate the tube at room temperature in the dark for 1 hour.
Add 20µl of 3M Sodium Acetate (pH 5.2) to the dye-coupled cDNA solution.
Add 500µl of Loading Buffer to the cDNA solution. Mix well by vortexing.
Place a SNAP Column onto a clear collection tube and load the cDNA/buffer solution.
Spin at 14,000g at room temperature for 1 minute; discard the flow-through.
Place the SNAP Column on the same collection tube; add 500µl of Wash Buffer to column.
Spin at 14,000g at room temperature for 1 minute; discard the flow-through.
Repeat Steps 3. 8-3.9 three times, for a total of three 500µl washes.
Spin one more time at 14,000g at room temperature for 1 minute; discard the flow-through.
Place the SNAP Column onto a new amber collection tube.
Add 65µl of warm DEPC-water (50°C) to the SNAP Column and incubate at room temperature for 1 minute.
Spin at 14,000*g* at room temperature for 1 minute and collect the flow-through. The flow-through should contain 60µl of your purified dye-coupled cDNA.

### Part 4: Assessing the Labeling Procedure.

Conduct a spectrophotometer assay to assess each Cy-cDNA labeling.  Calculate the total number of pmol of synthesized cDNA: [OD_260_ x vol. x 37ng/μl x 10^3^ng/pg]/324.5 pg/pmol. Calculate the total pmol of incorporated Cy-Dye for each labeling reaction: pmol Cy3 = [OD_550_ X vol.]/0.15; pmol Cy5 = [OD_650_ X vol.]/0.25.  Next, calculate the pmol of nucleotide/pmol dye ratio for each reaction.  Optimal reactions generate >6000pmol of cDNA (per 60ul), >200pmol of Cy-Dye (per 60ul), and a nucleotide/dye ratio that is <30.

### Part 5: Slide Pre-Wash

The PtGen2 Array is printed on Corning UltraGaps slides (amino-silane coated) and cDNAs are UV-cross linked.  Print spots are clearly visible due to the high salt content of the buffer.
Use a diamond tip pen to mark the slide for the upper and lower areas of the print area (optional).
Put approximately 250mL pre-warmed to 43°C 0.2% SDS solution into microscope slide wash dish (3” x 4”).
Place slides to be pre-hybridized in the slide rack and plunge into the SDS solution 15-20 times very vigorously to remove the salts on the slides.
Using forceps, carefully remove the slides from the rack and place them in a Coplin jar containing Pre-hybridization Buffer (5X SSC, 0.1% SDS, 1% BSA) that has been pre-warmed to 43°C.  Incubate at 43°C for at least one hour.

### Part 6: Pre-Hybridization

Set up five slide washing dishes, each filled with millipure dH_2_O, and one Coplin jar filled with molecular grade isopropanol.  Place the slides in a slide rack and process sequentially through five dishes by dunking 20 times each.
Remove slides two at a time from the fifth water wash and dip 5-6 times in the isopropanol and immediately place in slides into the slide microfuge (Chipmate benchtop, 2 positions) and spin 1 minute to dry.
Clean slides with compressed air filtered through a 0.22 μM filter cartridge and place them with the bar code facing up in a Genomic Solutions Hybridization Chamber.
Clean LifterSlips™ with filtered air and position them on the slides with the white strips facing down.  Use a yellow pipet tip to gently align the lifter slip on the slide (Washing LifterSlips™ first in 70% ethanol is optional.  We have found it to be unnecessary.)

### Part 7: Hybridization

*Should be carried out in the dark or low light.*

For each slide to be hybridized, calculate the amount of Cy-5 and Cy-3 labeled cDNA to give 50-75pmol of each target.  Combine those amounts into a single tube and dry in a vacuum desiccator with the heat setting at 37-45°C.  Avoid drying completely!
Resuspend the dried probes in 60mL hybridization buffer (made fresh that day) and vortex briefly.
Incubate the resuspended cDNAs for 5 minutes in a 95°C bath and then quick microfuge for 30 seconds.
Load the entire resuspended probe volume by slowly pipetting at the slide-LifterSlip™ junction at the bottom (bar code end) of the slide.  The slide will load by way of capillary action. (Some prefer to pipette on to the slide and then gently overlay the LifterSlip™ this is a one shot method since if you get bubbles you cannot move the LifterSlip™ after placement which can lead to spot smearing artifacts)
Place 20uL of 100mM DTT into each humidity well located at the ends of the chamber.
Place the top on the chamber and tighten the cam screws.  Cover the chamber with aluminum foil and immerse into a 48°C water bath for 12-18 hours with gentle agitation at 50-60 rpm.

### Part 8: Post-Hybridization Washes

*All washes should be carried out in the dark or low light.  Slides should never be allowed to dry until the final step.*

Set up one Coplin jar filled with Wash Solution #1 @ 53°C, and four slide washing dishes filled with 250mL of each of the following: 1) Wash Solution #1 @ 53°C , 2) Wash Solution #2 @ room temperature, 3 & 4) two dishes containing Wash Solution #3 @ room temperature.
Carefully remove the slides from the hybridization chambers and place them two at a time into the Coplin jar.  Leave for 30 seconds, then lift the slide out slowly and the lifter slip will remain in the jar.  Place slides in the slide rack and immediately immerse into Wash Solution #1 (1X SSC, 0.2% SDS, preheat to 43°C).  Repeat until all LifterSlips are removed and all slides to be washed are in the Wash Solution #1 dish. Plunge rack up and down 10 times vigorously.
Place dish in 53°C in air shaker with shaking at 50-100 rpm for 10 minutes.
Transfer rack to Wash Solution #2 (0.1X SSC, 0.2% SDS), plunge 10 times, and incubate at room temperature, slow shaking for 10 minutes.
Dip rack 15 times into the first Wash Solution #3 (0.1X SSC) to remove any residual SDS carry over from the Wash Solution #2.  Place rack in the second Wash Solution #3 dish, plunge 10 times, and then slow shaking for 10 minutes.
Place slides in 50mL Falcon capped tubes and spin @ 1,500 rpm in centrifuge equipped with a swinging bucket rotor. Remove slides to light proofed container, we use another Falcon tube covered with aluminum foil, and purge air from the tube with nitrogen gas, cap tightly (this reduces oxidation of the Cy dyes, especially Cy-5 in the summer when ozone levels are highest).  Slides can be stored for a few hours prior to scanning in the nitrogen purged tubes; however, best signal results are obtained when scanning is done immediately after washing.

### Part 9:  Data Collection and Statistical Analysis

Scan image data is collected on a PerkinElmer ScanArray.  We use a 10 micron scan setting and balance Cy3 and Cy5 signals using the line adjustment method as per the manufacturer’s instructions.
TIF files are gridded and raw signal data collected and filtered with ImaGene Version 7.5  (BioDiscovery, Inc., El Segundo, CA)
Data normalization and evaluation to determine statistically valid differentially expressed species is done with BRB Array tools Ver. 3.7.
Pattern analysis to facilitate determination of coordinately expressed genes is done with Gene Expression Pattern Analysis Software (GEPAS) Ver. 3.1.

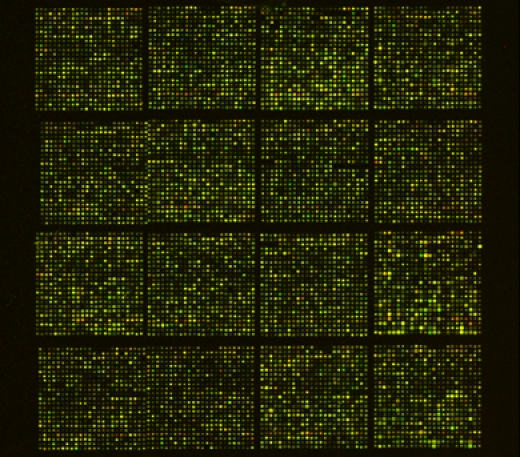


**Figure 1.** Example of PtGen2 array processed using this protoco**l.**


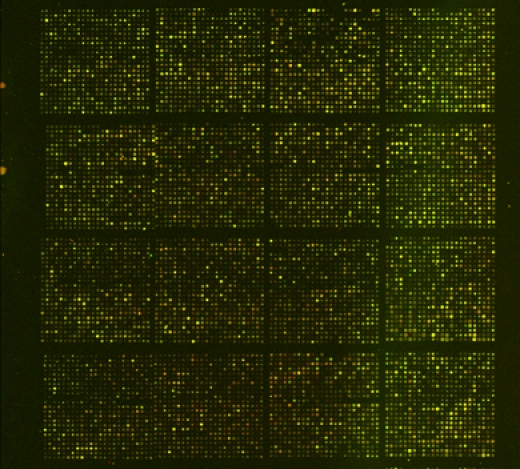


**Figure 2.** Example of PtGen2 array processed using standard hybridization and washing protocols.

## Discussion

PtGen2 is a recently developed, custom cDNA microarray that was designed to be used primarily by the loblolly pine research community.  Preliminary results generated thus far have shown the array to be a valuable tool in the evaluation of transcriptional events that occur in response to drought stress, as well as in monitoring changes that occur during embryo development in maritime pine, *Pinus pinaster*^ 5,6^ We have also recently demonstrated that PtGen2 works well in cross species hybridizations using target samples isolated from a diverse range of conifer genera.^5^ As PtGen2 becomes more utilized by researchers studying  gene expression in *Pinus* and other conifer species, this training video should provide them with the necessary technical foundation to generate quality and reproducible data.  In our hands, PtGen2 yielded better results when processed by more stringent hybridization and washing protocols than those typically employed in microarray work.  Other approaches to processing the PtGen2 array have been successful but lacked consistency.  Following the technical steps described here will help to increase consistency, particularly when processing large numbers of arrays, and will also help to greatly reduce and eliminate unwanted artifacts and high backgrounds.
